# Supplementary material for: Prevalence and predictors of prediabetes/type 2 diabetes mellitus among adolescents in the United States: NHANES (2021–2023)
Source: PLOS Glob Public Health. 2026 Feb 25;6(2):e0005596. doi: 10.1371/journal.pgph.0005596 (PMC12935269; doi:10.1371/journal.pgph.0005596)
Supplement: S1 Table — (DOCX) [file pgph.0005596.s002.docx]

**S1 Table.** Weighted prevalence of prediabetes and diabetes among US adolescents (10–19 years) in the NHANES fasting subsample (n = 571), stratified by key demographic and clinical variables.

| **Variable** | **Normal glucose**  **Weighted % (95% CI)** | **Prediabetes/Diabetes Weighted % (95% CI)** |
| --- | --- | --- |
| Overall Prevalence | 76.4 [70.4 – 82.4] | 23.6 [17.6 – 29.6] |
| **Gender** |  |  |
| Male | 69.73 [57.41 – 82.04] | 30.27 [17.96 – 2.59] |
| Female | 83.38 [77.53 – 89.22] | 16.62 [10.78 – 22.47] |
| **Race/Ethnicity** |  |  |
| Mexican American | 75.32 [65.75 – 84.88] | 24.68 [15.17 – 34.25] |
| Other Hispanic | 62.59 [33.71 – 91.47] | 37.41 [8.53 – 66.29] |
| Non-Hispanic White | 78.24 [71.41 – 85.06] | 21.76 [14.94 – 28.59] |
| Non-Hispanic Black | 82.33 [71.08 – 93.58] | 17.67 [6.42 – 28.92] |
| Non-Hispanic Asian | 86.998 [74.22 – 99.78] | 13.00 [0.22 – 25.78] |
| Other Race | 69.51 [52.50 – 86.53] | 30.49 [13.47 – 47.50] |
| **BMI Categories** |  |  |
| Underweight / healthy weight | 81.01 [75.71 – 86.31] | 18.99 [13.69 – 24.29] |
| Overweight / obesity | 68.49 [58.95 – 78.04] | 31.51 [21.96 – 41.05] |
| **Waist-to-Height Ratio** |  |  |
| Healthy (<0.5) | 81.67 [76.75 – 86.59] | 18.33 [13.41 – 23.25] |
| Abdominal obesity (≥ 0.5) | 68.68 [58.53 – 78.83] | 31.32 [21.17 – 41.47] |
| **Poverty income ratio (PIR)** |  |  |
| Low income (PIR<1.3) | 76.06 [66.80 – 85.33] | 23.94 [14.67 – 33.20] |
| Middle income (PIR ≥1.3 & <3.5) | 80.21 [68.65 – 91.77] | 19.79 [8.23 – 31.35] |
| High income (PIR≥3.5) | 72.38 [64.47 – 80.29] | 27.62 [19.71 – 35.53] |
| **Health Insurance Status** |  |  |
| Yes | 76.61 [71.07 – 82.14] | 23.39 [17.86 – 28.93] |
| No | 74.01 [37.07 – 110.95] | 25.99 [-10.95 – 62.93] |

*Weighted prevalence estimates were calculated using NHANES fasting subsample weight (WTSFA2YR), accounting for survey strata (SDMVSTRA) and primary sampling units (SDMVPSU); only participants with fasting glucose measurements (n = 571) were included; estimates are nationally representative of US adolescents.*
