# Supplementary material for: Prevalence and predictors of prediabetes/type 2 diabetes mellitus among adolescents in the United States: NHANES (2021–2023)
Source: PLOS Glob Public Health. 2026 Feb 25;6(2):e0005596. doi: 10.1371/journal.pgph.0005596 (PMC12935269; doi:10.1371/journal.pgph.0005596)
Supplement: S2 Table — (DOCX) [file pgph.0005596.s003.docx]

**S2 Table**: Sequential Logistic Regression Models Examining the Association Between Overweight/Obesity and Abnormal Glucose Status Among U.S. Adolescents (NHANES 2021–2023, MI = 20)

| **Variable** | **Model 1: Unadjusted OR (95% CI)** | **Model 2: + Socio-demographics & Waist/Height** | **Model 3: + Physical Activity & Sedentary** | **Model 4: + Dietary & Metabolic Markers** |
| --- | --- | --- | --- | --- |
| Overweight/Obesity | 1.57 (1.11–2.21)* | 0.76 (0.44–1.32) | 0.77 (0.45–1.33) | 0.74 (0.43–1.29) |
| Age (years) | — | 0.92 (0.85–0.99)* | 0.93 (0.86–1.00)* | 0.91 (0.83–0.99)* |
| Female (vs. male) | — | 0.45 (0.32–0.64)*** | 0.46 (0.32–0.65)*** | 0.53 (0.36–0.78)** |
| Waist-to-height ratio | — | 121.16 (5.25–2794.12)** | 131.01 (5.31–3230.68)** | 146.42 (5.39–3976.30)** |
| Met physical activity guidelines (≥60 Min * 7 days) | — | — | 1.09 (0.68–1.74) | 1.07 (0.66–1.71) |
| Sedentary ≥2 h/day | — | — | 0.82 (0.43–1.56) | 0.80 (0.42–1.52) |
| Energy intake (Within recommended, k/Cal) | — | — | — | 1.03 (0.68–1.57) |
| Energy intake (Above recommended, k/Cal) | — | — | — | 0.93 (0.48–1.81) |
| Total sugar intake (gm) | — | — | — | 1.00 (0.998–1.006) |
| Total cholesterol ≥170 mg/dL | — | — | — | 1.07 (0.72–1.59) |
| HDL ≤45 mg/dL | — | — | — | 1.05 (0.75–1.48) |
| hs-CRP (mg/L) | — | — | — | 0.99 (0.95–1.02) |
| Systolic BP (mmHg) | — | — | — | 1.02 (1.00–1.04) |
| Diastolic BP (mmHg) | — | — | — | 1.01 (0.98–1.03) |
| Model F test (p-value) | 6.88 (0.012)* | 3.49 (0.0001)*** | 2.89 (0.0003)*** | 2.22 (0.0010)*** |
| Number of imputations | 20 | 20 | 20 | 20 |
| N (imputed) | 1,998 | 1,998 | 1,998 | 1,998 |

*Notes: *p < 0.05, **p < 0.01, ***p < 0.001. Outcome: abnormal glucose status (glustatus_dichot). Models were sequentially adjusted as described in the Methods. All models were estimated using multiple imputation (20 datasets) and accounted for the NHANES survey design.*
