# Supplementary material for: Prevalence and predictors of prediabetes/type 2 diabetes mellitus among adolescents in the United States: NHANES (2021–2023)
Source: PLOS Glob Public Health. 2026 Feb 25;6(2):e0005596. doi: 10.1371/journal.pgph.0005596 (PMC12935269; doi:10.1371/journal.pgph.0005596)
Supplement: Attachment — Submitted filename: Response to reviewers.docx [file pgph.0005596.s005.docx]

**REVIEWERS COMMENTS**

**Response to Reviewer #1**

I sincerely thank the reviewer for the thoughtful and constructive feedback, which has improved the clarity and rigor of my manuscript. I address each comment below.

1. *Grouping prediabetes/diabetes***:** I agree that combining prediabetes and diabetes could obscure differences in risk factors. However, in my sample, diabetes was extremely rare (0.25% of adolescents; 5 out of1998 participants), making separate analyses statistically unreliable. Therefore, combining prediabetes and diabetes into a single outcome was necessary to maintain sufficient power and ensure stable estimates. This rationale has been clarified in the Methods section.
2. *Sensitivity analysis for separate outcomes:* As noted above, separate analyses for prediabetes and diabetes were not feasible due to the extremely low number of diabetes cases. Table presented as supplementary file.
3. *Energy intake* was categorized based on the Dietary Guidelines for Americans, 2020–2025, which recommend 1,400–2,200 kcal/day for females aged 10–13 years, 1,800–2,400 kcal/day for females aged 14–18 years, 1,600–2,600 kcal/day for males aged 9–13 years, and 2,000–3,200 kcal/day for males aged 14–18 years. Participants were classified as consuming (1) below, (2) within, or (3) above the recommended intake for their age and sex. This approach ensures that energy intake is evaluated relative to age- and sex-specific recommendations rather than arbitrary quartiles (U.S. Department of Agriculture and U.S. Department of Health and Human Services, 2020).
4. *Elevated BP definition:* The definition has been corrected to align with current American Academy of Pediatrics recommendations. That is, for adolescents aged 10–12 years: Normal BP: <90th percentile; Elevated BP: ≥90th to <95th percentile or 120/80 mmHg to <95th percentile (whichever is lower); Hypertension: ≥95th percentile to <95th percentile + 12 mmHg or ≥130/80 mmHg (whichever is lower). For adolescents aged ≥13 years: Normal BP: <120/<80 mmHg; Elevated BP: 120/<80 to 129/<80 mmHg; Hypertension: ≥130/80 mmHg.
5. *Collinearity/VIF reporting:* Information on collinearity and variance inflation factors has been moved to the Results section under a “Modeling Assumptions” subsection.

**Results:**

1. *Reliance on p-values:* Tables and text have been revised to emphasize effect sizes and confidence intervals rather than p-values, in accordance with ASA and ICMJE guidelines.
2. *Baseline demographics:* P-values in Table 1 have been removed; descriptive statistics are now reported as means (SD) or proportions.
3. *Table formatting:* Statistically significant values are no longer bolded in tables to avoid misinterpretation.
4. Weighting clarification: NHANES sampling weights, strata, and PSU variables were applied in all analyses to produce nationally representative estimates. This has been explicitly stated in the Methods.
5. Reporting prevalence differences: Differences between groups are now reported using prevalence estimates with 95% CIs rather than p-values. For example, adolescents with overweight/obesity showed a prevalence of abnormal glucose status of 36.4%, compared to 28.7% in healthy-weight adolescents.
6. Logistic regression reporting: All regression results now report odds ratios with 95% confidence intervals; p-values are provided only for reference.
7. BMI effect reversal and collider bias: Sensitivity analyses were conducted to explore potential confounding and collider bias. Sequentially adding covariates, including socio-demographics, waist-to-height ratio, physical activity, sedentary behavior, dietary intake, and metabolic markers, attenuated the association of BMI with abnormal glucose status, rendering it non-significant. This suggests that the observed relationship is influenced by demographic and metabolic factors, consistent with potential collider effects. Details are provided in Supplementary Table 2.

**Response to Reviewer #2:**

1. I thank the reviewer for the thoughtful and accurate summary of our study and findings. I appreciate the recognition of the high prevalence of prediabetes/diabetes and the strong association with central adiposity among U.S. adolescents. In response, I have further clarified the presentation of the results in the Results and Discussion sections to ensure that effect sizes, directions of association, and adjusted model findings are more clearly delineated.

Specifically, I have emphasized that while the odds ratio for elevated waist-to-height ratio was notably high, this likely reflects sparse data within certain strata and should be interpreted cautiously as an indication of a strong association rather than a literal increase in odds. I also revised the conclusion to underscore that the findings demonstrate associations, not causal relationships, consistent with the cross-sectional nature of NHANES data.
